# Dynamic roles of neutrophil extracellular traps in cancer cell adhesion and activation of Notch 1-mediated epithelial-to-mesenchymal transition in EGFR-driven lung cancer cells

**DOI:** 10.3389/fimmu.2024.1470620

**Published:** 2024-10-04

**Authors:** Jelena Dimitrov, Maurizio Maddalena, Cristina Terlizzi, Giovanna G. Altobelli, Sara Pellegrino, Tayyaba Mehmood, Viviana De Rosa, Francesca Iommelli, Silvana Del Vecchio

**Affiliations:** ^1^ Department of Advanced Biomedical Sciences, University “Federico II”, Naples, Italy; ^2^ Institute of Biostructures and Bioimaging, National Research Council, Naples, Italy

**Keywords:** neutrophil extracellular traps, epithelial mesenchymal transition, Notch 1, adhesion, migration, tumor microenvironment, metastasis

## Abstract

**Introduction:**

Neutrophil extracellular traps (NETs) are complex structures released by activated neutrophils that may modulate different steps of the metastatic cascade. The aim of our study was to investigate how NETs can modulate the adhesion properties of cancer cells and whether cell exposure to NETs can activate the epithelial-to-mesenchymal transition (EMT) program thus enhancing the migratory and invasive properties of tumor cells.

**Materials and methods:**

Different cancer cell lines were subjected to a solid-phase adhesion assay using NET-coated plates with or without the addition of antibodies against α5β1 or CCDC25 receptor. After 1-4 h of incubation, adherent cells were expressed as the percentage of total cell number. To test EMT occurrence, cells were treated with NETs for up to 48 h and then the levels of E-cadherin, vimentin, Snail, Slug, Zeb 1 and Twist 1 along with levels of Notch 1 and cleaved Notch 1 were determined by western blotting. Untreated and NET-treated cells were subjected to migration assays using 24-multiwell plates with transwell and FBS as chemoattractant.

**Results:**

Cancer cell adhesion to NET-coated plates varied between 30% and 92.7% and was significantly higher than that obtained in uncoated plates. The addition of antibodies against α5β1 or CCDC25 caused a strong reduction of cell adhesion to NETs. The prolonged exposure of EGFR-driven cancer cell lines to NETs caused the activation of the EMT program through the upregulation and cleavage of Notch 1 and was confirmed by the enhanced expression of EMT markers. The consequent loss of the epithelial phenotype induced a strong reduction of the expression of the oncogene driver. Cell migration was significantly enhanced in NET-treated cells as compared to untreated cells.

**Discussion:**

Our findings reveal the dynamic role of NETs that may provide a DNA and fibronectin rich environment for binding of many cancer cells at distant sites where the prolonged exposure to NETs triggers the EMT through the activation of Notch 1 signaling pathway with the subsequent enhancement of migratory and invasive properties of cancer cells. Furthermore, our findings provide an example of how an immune/inflammatory microenvironment may directly modulate the sensitivity of cancer cells to oncogene targeted agents.

## 1 Introduction

Neutrophil extracellular traps (NETs) are complex structures released by activated neutrophils that contain nucleic acids, histones and several proteases such as myeloperoxidase (MPO) and neutrophil elastase (NE) (1). The main role of NETs is in the innate immune response since they are able to entrap and kill pathogens (2, 3). However, in the last decade, a growing body of evidences indicates that NETs are involved in a number of different biologic processes including wound healing, autoimmunity, thrombotic disease and cancer progression (4, 5). In particular, NETs are reported to promote metastatic dissemination of cancer cells by entrapment of circulating tumor cells at distant sites (6, 7). In fact, deposition of NETs within hepatic sinusoidal spaces was associated with increased formation of hepatic micrometastases and subsequent development of gross metastatic lesions upon intrasplenic injection of cancer cells (6). In addition, it became increasingly clear that the role of NETs in promoting metastatic dissemination is not limited to a mechanical entrapment of circulating cancer cells but they are reported to promote cancer cell adhesion, proliferation, migration, and invasion through not completely elucidated signaling pathways (8–10).

In the effort to identify such signaling pathways, we showed that α5β1 and ανβ3 integrins mediate cancer cell adhesion to NETs by binding to their common substrate fibronectin, which was found to localize inside the web-like structure of NETs (8). Furthermore, by testing different cancer cell lines, we found that high levels of α5β1, ανβ3 and ανβ5 enhance cell adhesion to NETs whereas low levels of α5β1 prevents cell attachment to NETs (9). Other authors reported that the expression of β1 integrin on both cancer cells and NETs is important for the adhesion of circulating cancer cells to NETs (11) Furthermore, in a recent study, NET-DNA was shown to exert a chemotactic function by interacting with a DNA receptor, termed CCDC25, expressed on the plasma membrane of cancer cells (12). Following interaction with NET-DNA, this receptor was reported to activate integrin-linked kinase (ILK) that in turn recruits β-parvin and initiates the RAC1–CDC42 cascade to induce cytoskeleton rearrangement and directional migration of tumor cells.

In addition to their role in mediating cell adhesion and migration, some authors reported that NETs can induce activation of epithelial-mesenchymal transition (EMT) program in both normal and malignant cells (13–15). However, it is unclear whether this is a general property of NETs that can occur in all malignant cells independently from the context and whether it will require activation of specific signaling pathways or the contribution of multiple components of immune/inflammatory microenvironment. Some evidences indicate the involvement of TGF-β signaling in NET-induced metastatic spread of gastric cancer (15). Similarly, NF-kB/NLRP3 signaling was reported to be involved in NET-dependent lung cancer metastasis formation (16) and TLR9 signaling was considered important for the NET-dependent progression of diffuse large B cell lymphoma (17). However, the complex signaling landscape triggered by NETs upon binding to malignant cells is far from being fully elucidated.

Based on these considerations, we decided to test whether NETs can induce EMT in different cancer cell lines and whether the signaling pathways involved may be the same. In doing that we started from the notion that NETs can bind to α5β1 integrin and CCDC25 receptor, both expressed on the surface of cancer cells, and evaluate NET-dependent cell adhesion in several cancer cell lines. Then, we tested those cells for the expression of EMT markers after NET exposure for up to 48 hours and analyzed the molecular mechanisms and signaling mediators involved in NET-driven EMT.

## 2 Materials and methods

### 2.1 Production of NETs

Cell-free suspensions of NETs were obtained from differentiated human acute promyelocytic leukemia HL-60 cell line (ATCC Cat# CCL-240, RRID: CVCL_0002). Briefly, HL-60 cells were maintained in IMDM supplemented with 10% FBS. Differentiation of HL-60 cells into neutrophil-like cells was achieved by adding 1.3% dimethylsulfoxide (DMSO) to IMDM for 7 days as described previously (8). The differentiation was confirmed by the evaluation of the expression of neutrophil markers CD11b (Agilent Cat# R084101, RRID: AB_579547) and CD177 (Miltenyi Biotec Cat# 130-126-423, RRID: AB_2889477) by FACS analysis. For NETs production, differentiated HL-60 cells (dHL-60) were exposed to 25µM Calcium ionophore (A23187, Sigma-Aldrich, St. Louis, MO, USA) for 4 h in a humidified incubator at 37°C and 5% CO_2_. After exposure, the conditioned medium was recovered and centrifuged at 310xg for 10 min at 4°C to obtain a cell-free NETs-enriched supernatant. This supernatant was then centrifuged at 18000xg for 10 min at 4°C and the pellet containing NETs was resuspended in 100 µl of cold PBS. Measurement of double-stranded DNA concentration was performed using a NanoDrop ND-1000 spectrophotometer with V3.5.2 software (NanoDrop Technology, Cambridge, UK) and NET suspensions were stored at -80°C until used. Several NET preparations were needed to perform experiments in triplicates.

### 2.2 Characterization of cancer cell lines

Several tumor cell lines were selected for testing their response to NET exposure based on their origin, molecular profile and phenotypic features. In particular, lung cancer cell lines were selected for their EGFR or other oncogenes dependence whereas the criterion for selection of breast cancer cell lines was the presence of fully epithelial or partial mesenchymal features. Also, fibrosarcoma and glioblastoma cells were chosen because of their high metastatic potential and local invasion ability, respectively. Therefore, four non-small cell lung cancer cell lines, namely the two EGFR-driven HCC827 (ATCC Cat# CRL-2868, RRID: CVCL_2063) and H1975 (ATCC Cat# CRL-5908, RRID: CVCL_1511), the MET-driven H1993 (ATCC Cat# CRL-5909, RRID: CVCL_1512) and the KRAS-mutated A549 (ATCC Cat# CCL-185, RRID: CVCL_0023) cell lines were included in the study. HCC827 cells bear an activating deletion of exon 19 (delE746_A750) of EGFR (18). whereas H1975 cells present an activating point mutation in exon 21 (L858R) and also harbor the T790M mutation in the kinase domain of EGFR (19, 20), causing resistance to tyrosine kinase inhibitors. H1993 cells are reported to have a high level of MET gene amplification (15 copy numbers) (21, 22) and wild-type EGFR whereas A549 cell line is homozygous for c.34G>A/p.G12S KRAS mutation (23, 24). Furthermore, MDA-MB-231 (ATCC Cat# CRM-HTB-26, RRID: CVCL_0062) is a triple-negative breast cancer cell line with very aggressive and invasive behavior that is characterized by an enrichment of epithelial-mesenchymal transition markers whereas MCF-7 (ATCC Cat# HTB-22, RRID: CVCL_0031) breast cancer cells express estrogen and progesterone receptors and do not have amplification of HER2. Moreover, highly invasive human fibrosarcoma HT-1080 (ATCC Cat# CCL-121, RRID: CVCL_0317) cells and human glioblastoma U87-MG (ATCC Cat# HTB-14, RRID: CVCL_0022) cells were tested for response to NET exposure. Cancer cell lines H1975, HCC827, H1993 and MDA-MB-231 were maintained in complete RPMI 1640 supplemented with 10% FBS whereas MCF7, A549, HT1080 and U87-MG cells were grown in DMEM (Corning, Mediatech, Inc, Manassas, VA, USA) containing 10% FBS. All cell lines were cultured in a humidified incubator at 37°C and 5% CO_2_. Cells were preliminarly characterized for their expression of β1 chain of integrin family and CCDC25 receptor by western blotting and then subjected to solid phase adhesion assay in the presence or absence of antibodies against α5β1 integrin (see below) and CCDC25 (see below) receptor.

### 2.3 Cell adhesion assay

To test cell adhesion to NETs, a cell-free NET suspension was used to coat 24-well flat-bottomed plates. Each well was incubated overnight at 4°C with 5µg of NETs dissolved in 200µl of PBS. PBS and conditioned medium of dHL-60 were used as negative controls. After washing with cold PBS, each well was incubated with serum-free medium with 1% BSA for 1h at room temperature to inhibit non-specific binding. Then 3x10^5^ cells were seeded in each well and allowed to adhere for 1, 2 or 4 h in a humidified incubator at 37°C and 5% CO_2_. At the end of incubation, non-adherent cells were discarded and attached cells were counted expressing the results as a percentage of total cell number. At least three independent experiments were performed in duplicate for each cell line using different NET preparations. An additional negative control included pre-treatment of NET-coated wells with 10 μl of DNase I (10000 UI/ml, Roche, Mannheim, Germany) for 15 minutes at room temperature. To test the role of α5β1 integrin and CCDC25 receptor in promoting cell adhesion to NETs, 3×10^5^ cells were pre-incubated with 5μg/ml of blocking antibody recognizing α5β1 integrin (Millipore Cat# MAB2514, RRID: AB_94626) or CCDC25 (Thermo Fisher Scientific Cat# PA5-54735, RRID: AB_2639443) in 300μl of serum-free medium supplemented with 1% BSA for 1 h at 37°C and 5% CO_2_. Adhesion assay was then performed as previously described.

### 2.4 Cell treatment with NETs

To test whether cell exposure to NETs may activate the epithelial-to-mesenchymal transition program, cells were seeded in 6-well flat-bottomed plates at a density of 500000 per well in medium supplemented with 10% FBS and allowed to adhere overnight at 37°C and 5% CO_2_. After washing with cold PBS, adherent cells were incubated with 0.5 μg/ml of NET suspension in serum-free medium for 4 h, 24 h and 48 h in a humidified incubator and then lysed for western blot analysis. Levels of known markers of EMT were determined along with levels of potential signaling mediators downstream α5β1 integrin.

### 2.5 Immunoblot analysis

Whole-cell lysates were prepared as previously described (25, 26). Briefly, cells were lysed on ice in RIPA lysis buffer with protease and phosphatase inhibitors (Thermo Scientific Inc.). The suspension was then homogenized by passages through a 26-gauge needle and centrifuged at 16,000xg for 30 min at 4°C. The supernatant was collected, and protein concentration was determined by the Bradford assay (Bio-Rad). Western blot analysis of proteins from whole cell lysates was carried out using a standard procedure. Proteins were separated by gel electrophoresis and then transferred onto Polyvinylidene difluoride (PVDF) membranes. After blocking non-specific binding with 5% non-fat dry milk, PVDF membranes were probed by using primary antibodies recognizing E-cadherin (mouse monoclonal 1:1000; Santa Cruz Biotechnology Cat# sc-8426, RRID: AB_626780), vimentin (mouse monoclonal 1:1000; Abcam Cat# ab8069, RRID: AB_306239), fibronectin (rabbit polyclonal 1:1000; Thermo Fisher Scientific Cat# PA5-29578, RRID: AB_2547054), N-cadherin (mouse monoclonal 1:1000; Santa Cruz Biotechnology Cat# sc-59987, RRID: AB_781744), integrin β1 (mouse monoclonal 1:1000; Santa Cruz Biotechnology Cat# sc-13590, RRID: AB_627008), CCDC25 (mouse monoclonal 1:1000 Santa Cruz Biotechnology, Inc. #515201), GAPDH (rabbit monoclonal 1:1000; Cell Signaling Technology Cat# 2118, RRID: AB_561053), vinculin (mouse monoclonal, 1:1000; Santa Cruz Biotechnology Cat# sc-73614, RRID: AB_1131294), α tubulin (mouse monoclonal, 1:1000; Santa Cruz Biotechnology Cat# sc-5286, RRID: AB_628411), p-EGFR (rabbit monoclonal, 1:1000; Cell Signaling Technology Cat# 3777, RRID: AB_2096270), EGFR (mouse monoclonal, 1:1000; Santa Cruz Biotechnology Cat# sc-373746, RRID: AB_10920395), p-AKT (mouse monoclonal, 1:1000; Cell Signaling Technology Cat# 4051, RRID: AB_331158), AKT (rabbit, polyclonal; 1:1000; Cell Signaling Technology Cat# 9272, RRID: AB_329827), phospho-p44/42 MAPK (ERK 1/2) (rabbit polyclonal; 1:1000; Cell Signaling Technology Cat# 9101, RRID: AB_331646), p44/42 MAPK (ERK 1/2) (rabbit polyclonal; Cell Signaling Technology Cat# 9102, RRID: AB_330744), cyclin D1 (rabbit polyclonal; 1:500; Cell Signaling Technology Cat# 2922, RRID: AB_2228523), p-ILK (rabbit polyclonal, 1:1000; Millipore Cat# AB1076, RRID: AB_10807157), ILK (mouse monoclonal; 1:500; Santa Cruz Biotechnology Cat# sc-20019, RRID: AB_627807), CDC42 (rabbit polyclonal, 1:1000; Millipore Cat# 07-1466, RRID: AB_1977123) SNAI1 (rabbit polyclonal; 1:1000; Millipore Cat# ABD38, RRID: AB_11213147), ZEB1 (rabbit polyclonal; 1:1000; Sigma-Aldrich Cat# SAB3500514, RRID: AB_10896384), SLUG (SNAI2) (rabbit polyclonal; 1:1000; Merck; #ABE993), TWIST 1 (rabbit polyclonal, 1:1000; Sigma-Aldrich Cat# T6451, RRID: AB_609890), Notch 1 (rabbit monoclonal, 1:1000; Cell Signaling Technology Cat# 3608, RRID: AB_2153354) and Cleaved Notch 1 (rabbit monoclonal, 1:1000; Cell Signaling Technology Cat# 4147, RRID: AB_2153348). Secondary antibody Peroxidase-conjugated AffiniPure Goat Anti-Rabbit IgG (H+L) (Jackson ImmunoResearch Labs Cat# 111-035-144, RRID: AB_2307391) and Peroxidase-conjugated AffiniPure Goat Anti-Mouse IgG (H+L) (Jackson ImmunoResearch Labs Cat# 115-035-003, RRID: AB_10015289). A commercially available ECL kit (GE Healthcare) was used to reveal the reaction and images were obtained using the ChemiDoc imaging system (Image Lab, Bio-Rad). The western blotting signal of the proteins of interest was then quantified by morphodensitometric analysis using ImageJ software (NIH, Bethesda, MD,USA). The product of the area and the mean signal intensity of each band was determined and normalized to the same product obtained from the loading control band. The results are expressed as relative protein levels of each NET-treated sample compared to the corresponding untreated internal control.

### 2.6 Cell migration assay

The ability of NETs to promote migration of tumor cells was evaluated by performing migration assays in 24-well plates using transwell (Corning) with 8.0 µm pore insert. Briefly, we added 600µl of RPMI-1640 supplemented or not with 3% and 10% FBS as chemoattractant in the lower compartment and 5×10^4^ H1975 cells in 150 µl of serum-free RPMI-1640 were seeded in the upper compartment. Cells had been pre-treated for 24 h with 0.5 μg/ml of NETs or NETs digested with DNase I. Cells were allowed to migrate for 16 h at 37°C and 5% CO_2_. At the end of incubation non-migrated cells were removed from the upper chamber with a cotton swab, whereas cells that had migrated through the membrane were fixed with 70% ethanol, stained with crystal violet and counted. Five randomly chosen microscopic fields were selected for counting and results were expressed as mean number of cells per field. In parallel experiments, a free-cell NET suspension containing 5µg/ml and 15µg/ml of NETs in serum-free medium was used as chemoattractant in the lower compartment. In negative controls NETs were pre-treated with DNase I. Then, untreated H1975 cells were seeded in the upper compartment, allowed to migrate for 20 h at 37°C and 5% CO_2_ and counted as described above.

### 2.7 Statistics

The software MedCalc for Windows, version 10.3.2.0 (MedCalc Software, Mariakerke, Belgium) was used for statistical analysis. Means from unpaired data were compared by Student’s t-test. Differences among multiple groups were evaluated by analysis of variance (ANOVA) followed by pairwise comparisons. Chi-squared test was used for analysis of categorical data. A probability value of <0.05 was considered statistically significant.

## 3 Results

### 3.1 Characterization of tumor cell lines

**Figure 1:**
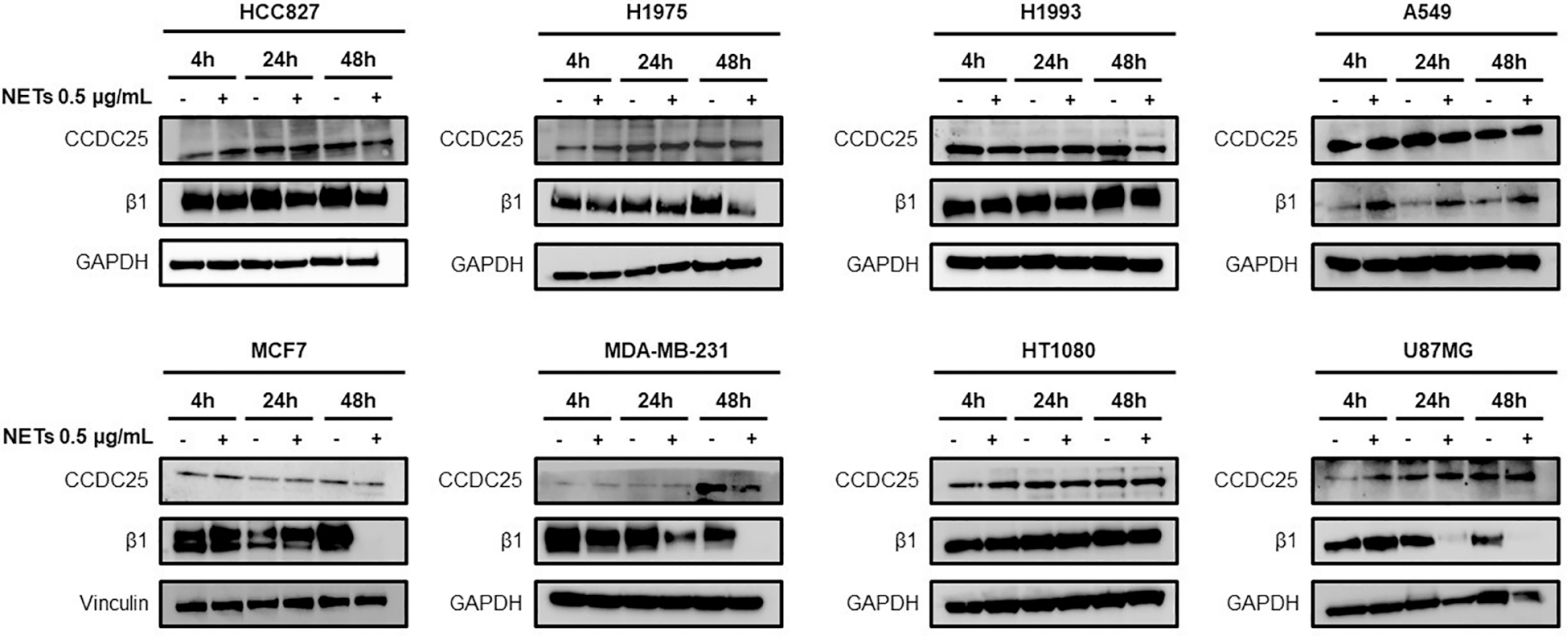
Levels of CCDC25 and β1 chain expression in different cancer cell lines. Cells were exposed or not to 0.5 µg/mL NETs for 4 h, 24 h and 48 h and then subjected to western blot analysis. GAPDH or vinculin were used as equal loading.

Different cancer cell lines were characterized for the expression of β1 chain and CCDC25 by western blotting in basal condition and after 4 h, 24 h and 48 h of cell exposure to NET suspension. **Figure 1** shows the levels of β1 chain and CCDC25 in basal conditions and in response to NET exposure. CCDC25 was expressed in all the cell lines tested and its levels remained substantially unchanged in response to NET exposure. Also, β1 chain was expressed in all the cell lines tested but its levels were strongly reduced in response to NET exposure in HCC827, H1975, MCF7, MDA-MB-231 and U87MG cells. The results of quantitative analysis of β1 chain and CCDC25 levels in HCC827 and H1975 cells are shown in **Supplementary Figure S1**.

### 3.2 NET-dependent adhesion of cancer cell lines

**Figure 2:**
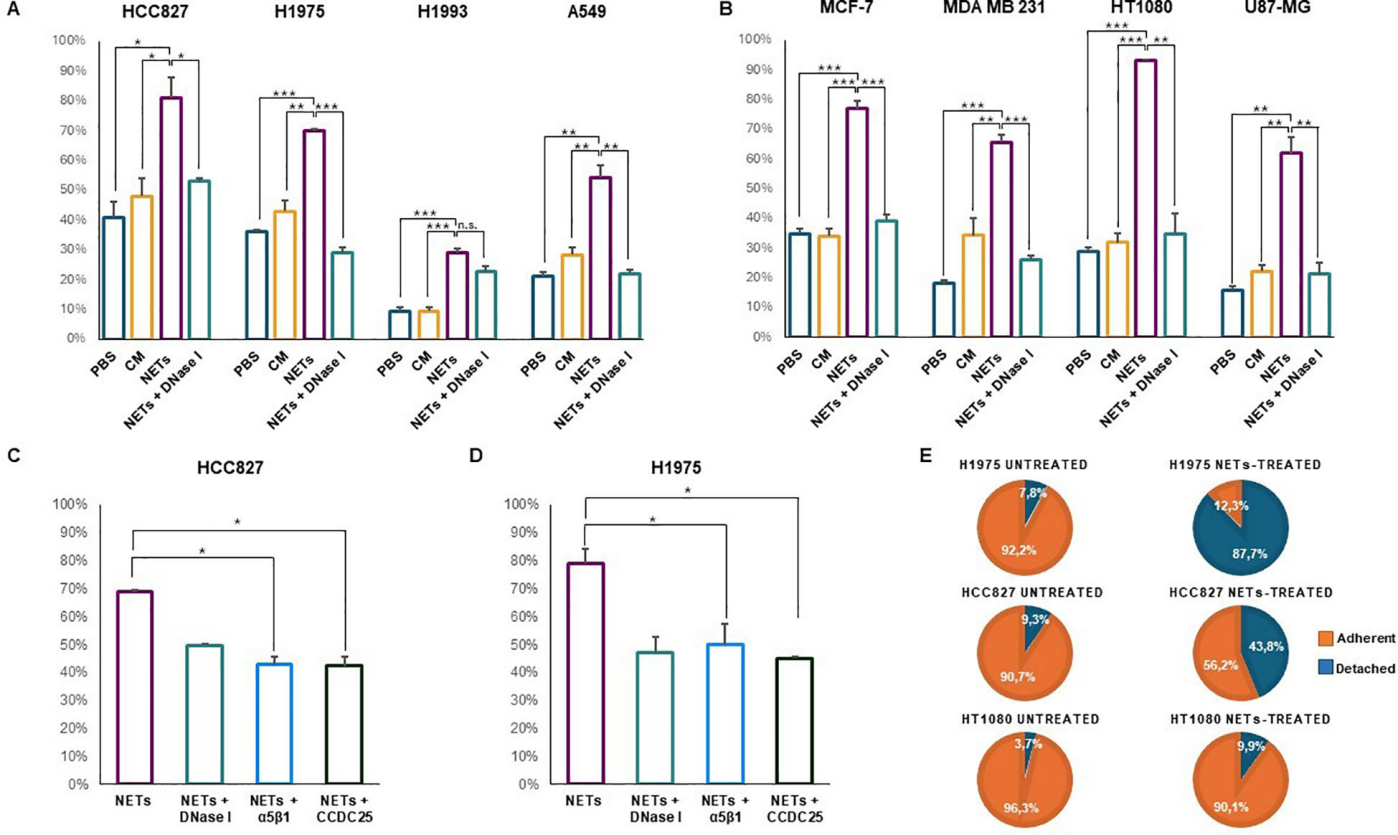
Cell adhesion of different cancer cell lines in the presence or absence of NETs. Cells were seeded and allowed to adhere to NET-coated multi-well plates for 1, 2 or 4 h depending on the cell line **(A–D)**. Negative controls were plates coated with PBS or conditioned medium of dHL-60 and NET-coated plates pre-incubated with DNase I. Results are expressed as percentage of total cell number in each well. In parallel experiments, adherent cells were exposed to 0.5 µg/mL NETs for 48 h followed by counting of adherent and detached viable cells **(E)**. **(A)** Cell adhesion (mean ± SE) obtained in HCC827 (2 h), H1975 (2 h), H1993 (2 h) and A549 (1 h) lung cancer cell lines. **(B)** Cell adhesion (mean ± SE) obtained in MCF7 (2 h) and MDA MB231 (2 h) breast cancer cells, HT1080 fibrosarcoma (1 h) and U87-MG glioblastoma (4 h) cell lines. **(C)** Cell adhesion (mean ± SE) to NET-coated plates obtained in HCC827 cells pre-incubated with blocking antibodies against α5β1 integrin and CCDC25 receptor. **(D)** Cell adhesion (mean ± SE) to NET-coated plates obtained in H1975 cells pre-incubated with blocking antibodies against α5β1 integrin and CCDC25 receptor. **(E)** Percentage of adherent and detached viable cells exposed or not to NETs in the medium for 48 h. Statistical significance: *p<0.05; **p< 0.01; ***p< 0.001; ns, not significant.

Cell adhesion to NETs was tested by incubating untreated cancer cell lines with NET-coated plates for 1, 2 or 4 h in a humidified incubator at 37°C and 5% CO_2_. Adherent cells were expressed as percentage of total cell number. PBS and conditioned medium of dHL-60 were used as negative controls along with pre-treatment of NET-coated wells with DNase I. **Figures 2A, B** show the results of solid-phase adhesion assay performed in all conditions for each cell line. Cell adhesion to NET-coated plates varied between 30% and 92.7% depending on the cell line and it was significantly higher than that obtained in uncoated plates pre-incubated with PBS and CM. Except for H1993 cell line, cell adhesion to NETs decreased significantly when NET-coated wells were subjected to pre-treatment with DNase I. P values for each condition and cell line are reported in **Figures 2A, B**. The different ability of cell lines to specifically adhere to NETs may depend on many factors including their origin, phenotypic features and especially molecular profile. The lowest percentage of cell adhesion to NETs was observed in H1993 cells that were characterized by the amplification of MET gene, a proto-oncogene encoding for the hepatocyte growth factor receptor that along with its ligand promotes cell motility and migration.

Since CCDC25 receptor was reported to bind the DNA component of NETs (12) and α5β1 integrin was reported to bind fibronectin included in the NET structure (8), we determined whether an excess of antibodies against CCDC25 and α5β1 could prevent cell adhesion to NETs. **Figures 2C, D** show that in HCC827 and H1975 cells both antibodies caused a statistically significant reduction of cell adhesion to NETs similar to that obtained with disruption of NET structures with DNase I. These findings indicate that both CCDC25 and α5β1 can be involved in cell binding to NETs.

Then we tested whether the prolonged incubation of adherent cells with NET suspensions may cause a reduction of cell adhesion. After 48 h of incubation with 0.5 μg/ml of NET suspension, we found that 87.7% of H1975 and 43.8% of HCC827 viable cells lost their adhesion to uncoated plates whereas only 7.8% of untreated H1975 cells (χ2 = 125, p< 0.0001) and 9.3% of untreated HCC827 cells (χ2 = 29.67, p< 0.0001) were detached at the same time point (**Figure 2E**). When we tested HT1080 cells that did not show any changes in the expression levels of β1 chain and CCDC25 after 48 h exposure to NETs, we found that 9.9% of NET-treated viable cells lost their adhesion to uncoated plates as compared to 3.7% of untreated cells (χ2 = 1.92, p = 0.1658). Therefore, when NETs are used as an adhesion substrate in a solid-phase adhesion assay, they promote cell attachment. However, the prolonged exposure to NETs may promote cell detachment.

### 3.3 NET-induced EMT in selected cancer cell lines

**Figure 3:**
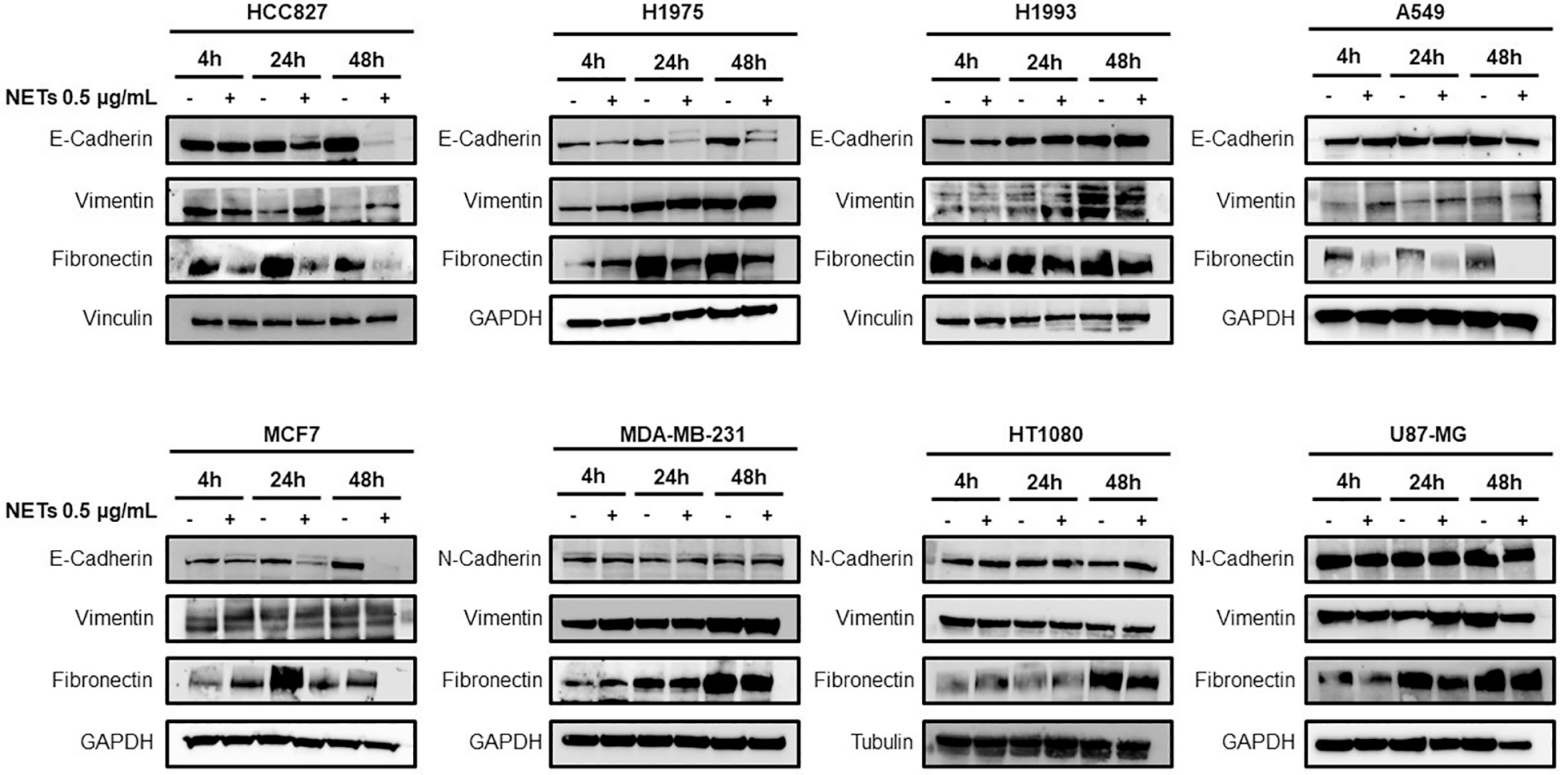
Expression of EMT markers in response to NET treatment. Levels of E-cadherin, vimentin, N-cadherin and fibronectin in different cancer cell lines exposed or not to 0.5 µg/mL NETs for 4 h, 24 h and 48 h. GADPH, vinculin and tubulin were used as equal loading.

Based on these observations, adherent cancer cells were incubated with 0.5 μg/ml of NET suspension in serum-free medium for 4 h, 24 h and 48 h and then tested for the expression of EMT markers by western blot analysis. **Figure 3** shows the levels of E-cadherin, vimentin and fibronectin in HCC827, H1975, H1993 and A549 lung cancer cell lines, MCF7 and MDA-MB-231 breast cancer cell lines and H1080 fibrosarcoma and U87-MG glioblastoma cells. Exposure to NETs caused a decrease of E-cadherin in HCC827, H1975 and MCF-7 cells and an increase of vimentin in HCC827 and H1975 cells indicating the activation of the EMT program in those cells. The results of quantitative analysis of E-cadherin and vimentin levels in HCC827 and H1975 cells are shown in **Supplementary Figure S2**. Similar findings, although less prominent, were found in A549 lung cancer cells. The same EMT markers were unchanged in untreated and NET-treated H1993 cells whereas MDA-MB231, HT1080 and U87-MG did not show significant changes in levels of N-cadherin and vimentin upon exposure to NETs. Fibronectin levels decreased in NET-treated HCC827, H1975, MCF-7 and A549 cells as compared to untreated control and have a variable pattern in the other cancer cell lines.

**Figure 4:**
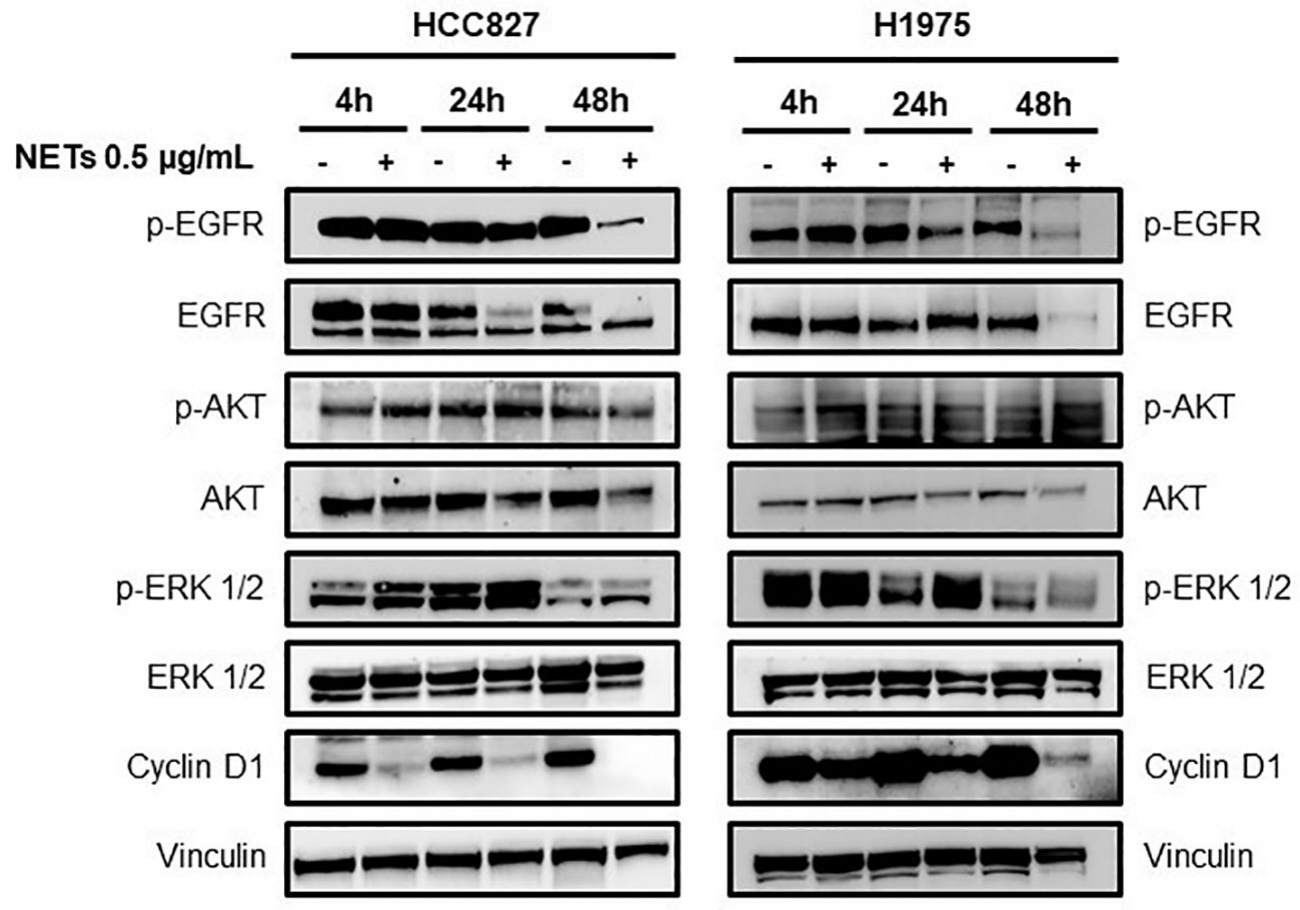
Loss of epithelial phenotype in EGFR-driven lung cancer cells exposed to NETs. HCC827 and H1975 cells were incubated with 0.5 µg/mL NETs for 4 h, 24 h and 48 h. Levels of p-EGFR, EGFR, p-AKT, AKT, p-ERK 1/2, ERK 1/2 and cyclin D1 were determined. Vinculin were used as equal loading.

The loss of the epithelial phenotype in HCC827 and H1975 cells was confirmed by the strong reduction of total and phosphorylated EGFR levels and by the downregulation of the EGFR downstream signaling pathway 48 h after NETs exposure (**Figure 4**). Interestingly levels of cyclin D1 indicating cell proliferation rate was decreased already at 4 h after exposure to NETs. Similarly, NET-treated MCF-7 showed a decrease of phosphorylated EGFR as compared to untreated control at 48 h (data not shown). These findings taken together indicate that exposure of EGFR-driven lung cancer cell lines to NETs induces the activation of the EMT program and loss of the epithelial phenotype.

### 3.4 NET-dependent enhancement of cell migration

**Figure 5:**
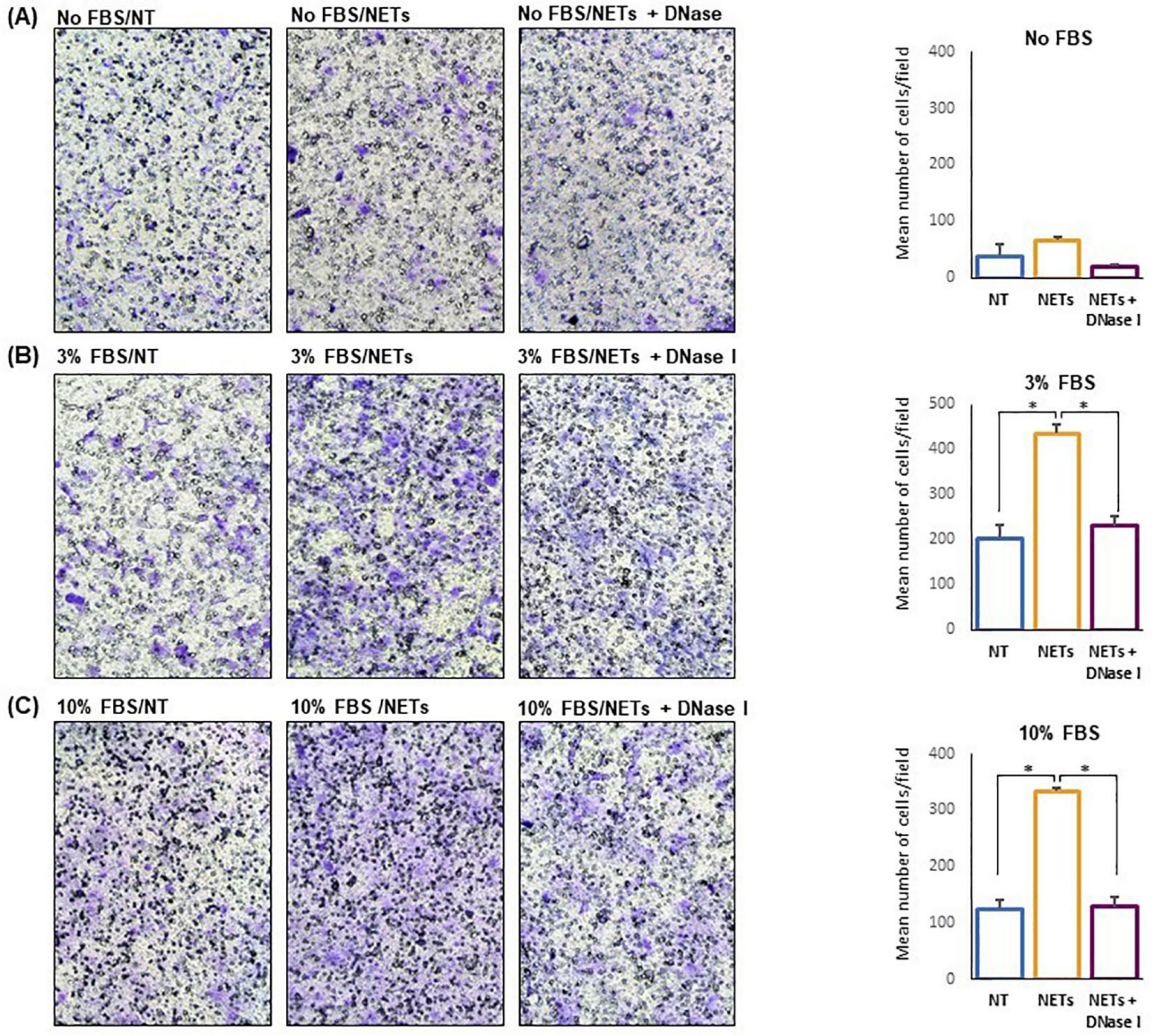
NET-dependent enhancement of cell migration. Untreated or NET-treated H1975 cells were seeded in the upper compartment of transwell and allowed to migrate for 16 h. No chemoattractant, 3% FBS or 10%FBS was added to the medium in the lower compartment. On the left, representative images showing migrated cells (100X Magnification) obtained with no FBS **(A)**, 3% FBS **(B)** and 10% FBS **(C)** in the lower compartment. On the right, the corresponding graph showing the mean number/field (± SE) of migrated cells obtained in each condition. Cell migration was 1.7 **(A)**, 2.1 **(B)**, and 2.7 **(C)** folds higher in NET-treated cells as compared to untreated cells when medium without serum, or supplemented with 3% FBS and 10% FBS, respectively, were used as chemoattractant. Statistical significance: *p<0.05.

Cell migration experiments were performed using 24-multiwell plates with transwell and FBS as chemoattractant (**Figure 5**). Untreated and NET-treated H1975 cells were seeded in the upper compartment and allowed to migrate. An additional negative control was represented by H1975 exposed to DNase treated NETs. When no chemoattractant was added in the lower compartment, migration of NET-treated cells was enhanced as compared to that of untreated cells although differences did not reach statistical significance (**Figure 5A**). When 3% FBS was added to medium and used as chemoattractant, cell migration was significantly different in the 3 groups (F-ratio= 28.036, p=0.01) with NET-treated cells reaching the highest values (**Figure 5B**). When the chemoattractant was 10% FBS added to medium, an even higher difference in the cell migration was observed between NET-treated cells and the other 2 groups (F-ratio=76.185, p=0.003) (**Figure 5C**). In fact, cell migration was 1.7, 2.1, and 2.7 folds higher in NET-treated cells as compared to untreated cells when no chemoattractant, 3% FBS and 10% FBS was used, respectively. Similar results were obtained when NET suspensions were used as chemoattractant. When using 15ug/ml of NETs, a 3-fold increase of cell migration was observed as compared to negative controls.

### 3.5 Signaling pathways involved in NET-driven EMT

**Figure 6:**
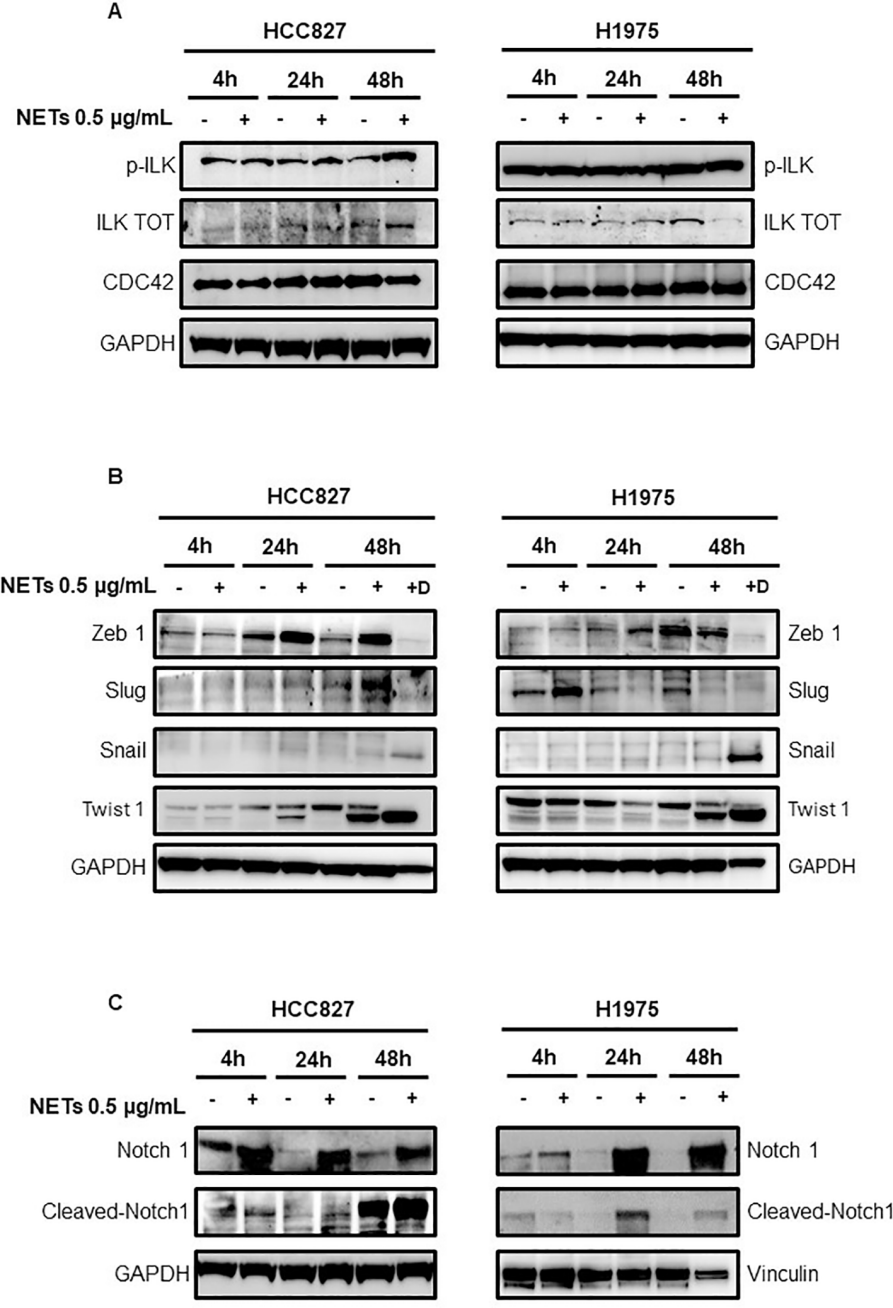
Levels of signaling mediators in EGFR-driven HCC827 and H1975 cells exposed to NETs. **(A)** Levels of p-ILK, ILK and CDC42 in cells exposed or not to 0.5 µg/mL NETs for 4 h, 24 h and 48 h. **(B)** Levels of transcription factors in adherent cells exposed or not to treatment with NETs for 4 h, 24 h and 48 h (lanes 2,4,6) and in viable cells that were detached after 48 h treatment with NETs (lane 7, +D). **(C)** Levels of total Notch 1 and cleaved Notch 1 in HCC827 and H1975 cells exposed or not to NETs for 4 h, 24 h and 48 h. GAPDH and vinculin were used as equal loading.

Since CCDC25 and α5β1 integrin are the main receptors mediating cell adhesion to NETs, we firstly tested the activation of their downstream pathways by determining the levels of total ILK, phosphorylated ILK and CDC42 in HCC827 and H1975 cells. A slight increase of phosphorylated and total ILK was found in HCC827 cells exposed to NETs as compared to untreated controls (**Figure 6A**). No significant changes of phosphorylated ILK were observed in NET-treated H1975 cells whereas total ILK levels were decreased. In both cell lines CDC42 levels were unchanged in response to NET treatment.

Then, the transcription factors Snail, Slug, Zeb 1 and Twist 1 were tested in adherent cells exposed to treatment with NETs for 4, 24 and 48 h (**Figure 6B**, lanes 2,4,6) and in cells that were detached after 48 h treatment with NETs (**Figure 6B**, lane 7 +D). Zeb1 expression was enhanced at 24 h in both cell lines and also at 48 h in HCC827 cells. Slug levels were increased at 48 h in HCC827 cells and at 4 h in H1975 cells. Levels of Snail were slightly increased at 24 h and 48 h in adherent cells and strongly upregulated in detached cells after 48 h treatment. Twist 1 was strongly enhanced in adherent and detached HCC827 and H1975 cells exposed to NETs for 48 h, and an increase was also observed at 24 h in treated HCC827 cells.

To clarify how signaling of EMT activation reaches transcription factors, we tested levels of total Notch 1 and cleaved Notch 1 in HCC827 and H1975 cells. We selected Notch 1 because it was reported to be one of the main drivers of the EMT program in several cancer cells and was up-regulated in H1975 cells when they grow as tumor spheres (27). **Figure 6C** shows an increase of total Notch 1 in HCC827 and H1975 cells at all time points and an increase of cleaved Notch 1 at 24 and 48 h post-treatment.

## 4 Discussion

Our study showed that NETs serve as an adhesion substrate for all the cell lines tested but the prolonged cancer cell exposure to NETs results in the activation of the EMT program in HCC827, H1975 and MCF7 cells with a subsequent enhancement of their migratory abilities. The dual role of NETs in promoting cell adhesion on one side and enhancing the migratory properties of cancer cells on the other side may reflect the dynamic of interactions of NETs in the peripheral vasculature of cancer patients. Circulating NETs may be retained in small blood vessels where they provide a DNA and fibronectin-rich microenvironment that can serve as a scaffold for the attachment of a large panel of cancer cells expressing CCDC25 receptor and β1 chain of integrin family. Prolonged binding to NETs can subsequently activate the EMT program enhancing the migratory abilities of cancer cells thus favouring the pre-metastatic niche formation. Similarly, circulating tumor cells that interact with NETs in suspension can acquire a mesenchymal phenotype that promotes their spread at distant organs.

Although certain cell lines have been reported to express EMT markers in response to NETs exposure (13–15), our study evaluated the activation of the EMT program in a large panel of cancer cell lines. We found that, while all cancer cell lines can bind to NETs, only few of them can activate the EMT program in response to prolonged exposure to NETs, at least at the dose level adopted in our study. This means that some cell lines are more prone to activate the EMT program due to the permissive status of their signaling network. To the best of our knowledge, our study was the first to demonstrate that Notch 1 was promptly upregulated in response to NETs and subsequently activated by cleavage. Therefore Notch 1 may have a central role in the NET-dependent induction of EMT especially in oncogene driven NSCLC. At present we do not know whether upregulation and activation of Notch 1 is triggered by a cross-talk with the integrin signaling cascade, by the close interaction between cells simultaneously interacting with NETs or by modulation through other signaling pathways such as TGF-β and NF-kB/NLRP3 (15, 16). It is also possible that cancer cells have redundant mechanisms to activate EMT in response to NETs. In any case, after 48 h treatment the transition is complete as shown by the expression levels of transcription factors.

The Notch 1-mediated activation of the EMT program implies the downregulation of EGFR signaling cascade with a strong reduction of the expression of oncogene driver leading to the consequent onset of cancer cell resistance to EGFR inhibitors. Our findings represent a clear example of how an immune/inflammatory microenvironment may directly modulate the sensitivity of cancer cells to oncogene targeted agents. A recent study in animal models of breast cancer reported that chemotherapy can induce neutrophil recruitment in lung metastases and the subsequent NET release can confer treatment resistance to chemotherapy via TGF-β activation (28). It would be interesting to know whether the TGF-β activation in metastatic breast cancer cells leads to Notch 1 upregulation. The interplay between cancer cells and neutrophils has been widely investigated and, depending on the context, neutrophils can either promote or inhibit cancer growth and progression. Here, the point is that neutrophils and probably other immune/inflammatory cells in the microenvironment can modulate tumor response to both targeted and cytotoxic cancer therapy.

Our study was the first to simultaneously test the role of β1 chain and CCDC25 receptor in cancer cell adhesion to NETs and we can state that these two molecules are equally effective in promoting cell adhesion. Therefore, cancer cells have redundant mechanisms of adhesion to NETs although it remains unclear whether one of the two receptors can trigger upregulation and activation of Notch 1 receptor. Previous studies reported the reciprocal interaction between integrins and Notch 1 pathways (29) and in particular between β1 integrin and Notch 1 (30) especially during tissue development. On the other hand, a non-canonical activation of Notch 1 can be induced by other signaling pathways in many cancer cells (31) and activation of ILK was reported to be upstream of Notch 1 pathway in leukemic cells (32). Additional studies by silencing β1chain or CCDC25 receptor may clarify the relative contribution of the two molecules to the activation of Notch 1.

In conclusion, our study showed that cancer cell adhesion to NETs can be associated to upregulation and activation of Notch 1 that in turn promotes activation of the epithelial-to-mesenchymal transition program thus enhancing the migratory and invasiveness of cancer cells at metastatic sites.

## Data Availability

The raw data supporting the conclusions of this article will be made available by the authors, without undue reservation.
