# Supplementary material for: Dynamic roles of neutrophil extracellular traps in cancer cell adhesion and activation of Notch 1-mediated epithelial-to-mesenchymal transition in EGFR-driven lung cancer cells
Source: Front Immunol. 2024 Oct 4;15:1470620. doi: 10.3389/fimmu.2024.1470620 (PMC11487346; doi:10.3389/fimmu.2024.1470620)
Supplement: Supplementary file 1 [file DataSheet1.docx]

Supplementary Material

## Supplementary Figures


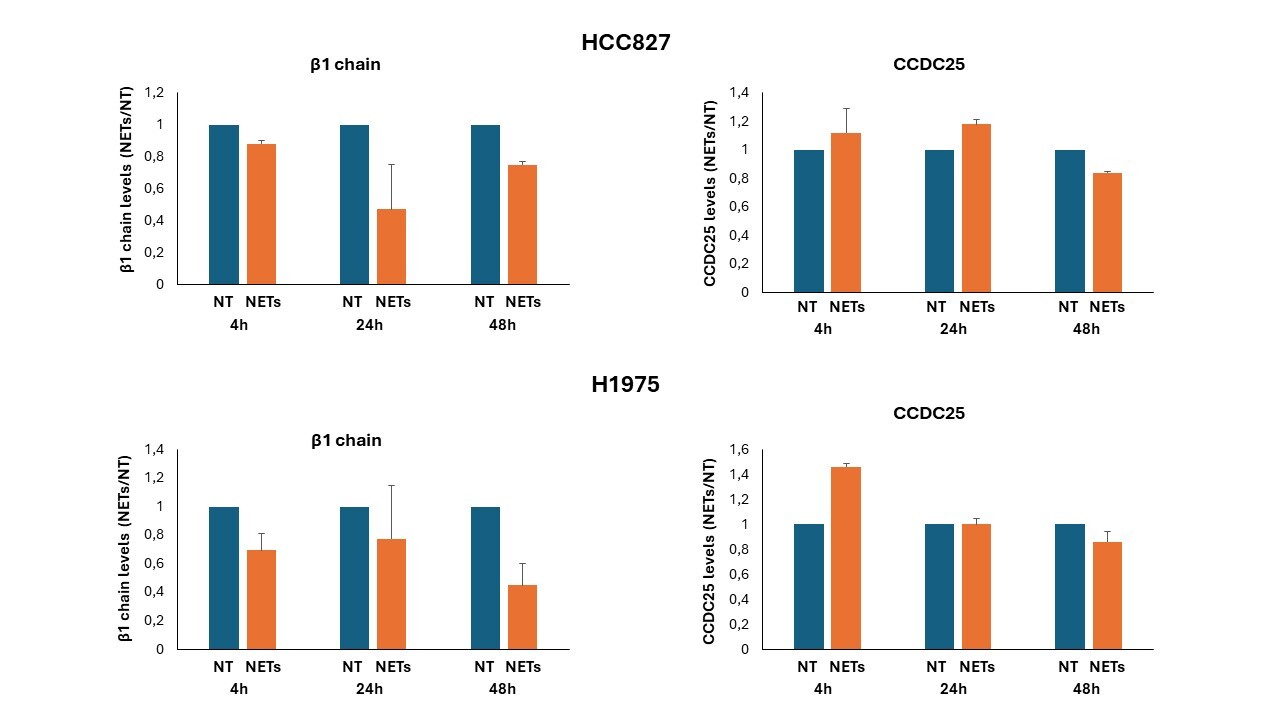


**Supplementary Figure 1.** Results of quantitative analysis of β1 chain and CCDC25 in HCC827 and H1975 cells. The data are expressed as the relative levels of protein in each NET-treated sample as compared to the corresponding untreated internal control. NET-treated samples showed up to a 60% reduction of β1 chain levels in both cell lines whereas CCDC25 levels showed fluctuations without a specific pattern.


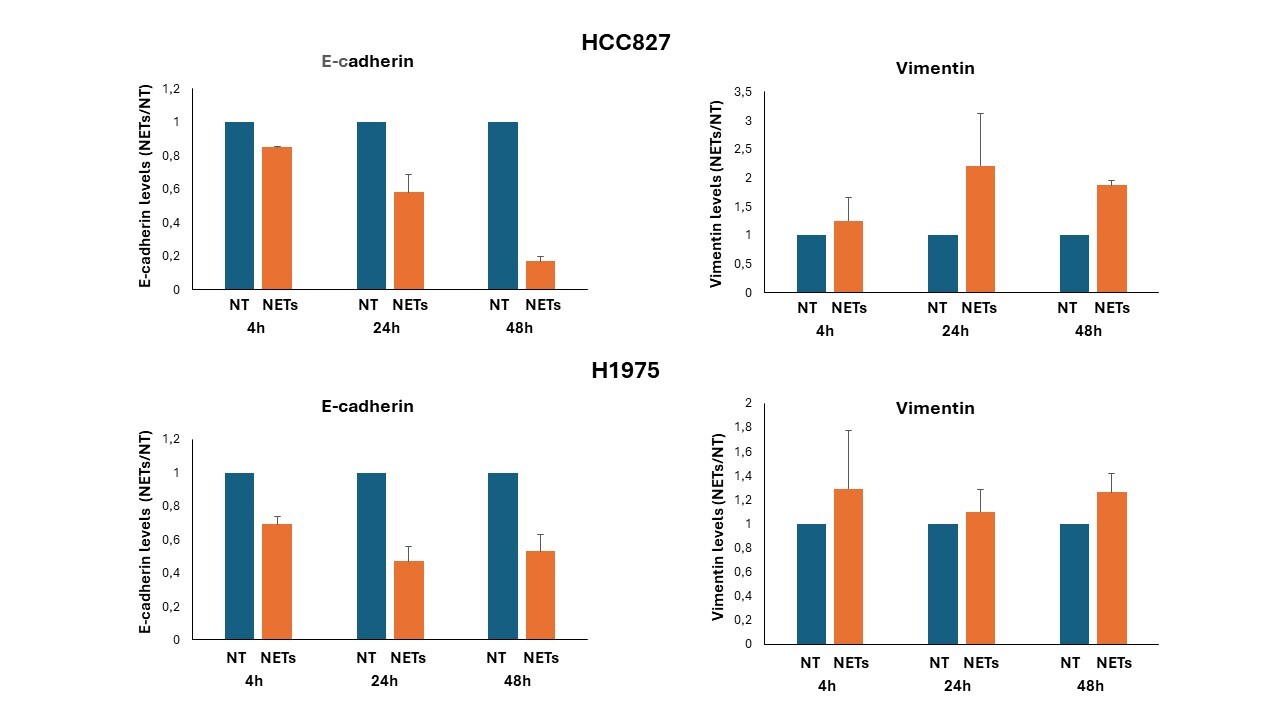


**Supplementary Figure 2**. Results of quantitative analysis of E-cadherin and vimentin in HCC827 and H1975 cells. The data are expressed as the relative levels of protein in each NET-treated sample as compared to the corresponding untreated internal control. NET-treated samples showed up to a 80% reduction of E-cadherin levels in both cell lines whereas vimentin levels were increased.
